# Human Pathogens in Primary Production Systems

**DOI:** 10.3390/microorganisms11030750

**Published:** 2023-03-14

**Authors:** Leo van Overbeek

**Affiliations:** Wageningen Plant Research, Wageningen University and Research, 6708 PB Wageningen, The Netherlands; leo.vanoverbeek@wur.nl

Human pathogenic micro-organisms can contaminate plants. Plants whose products can be consumed freshly or after minimal processing are of specific concern. It is under debate whether contaminations only occur at harvest or the after harvest processing of crops, or if they can already occur at the primary plant production stages.

Plants may be considered as secondary habitats for human pathogens [1], and, although they do not possess the full capacity to invade and colonize internal tissues of plants, like plant pathogens and endophytes do [2], they are still capable of maintaining themselves in the neighborhood of, and even inside, plants [3], and to proliferate in these ecosystems. Human pathogens can respond to chemical signals from plants [4] and, from that perspective, human pathogens may share properties with other micro-organisms commonly present in plant microbiomes. From an evolutionary perspective, it make sense that particular groups of zoonotic species are able to use plants as secondary habitats. These microbes can be transferred via feces among different flocks that graze on the same land [5]. Longer persistence on grazed plants may contribute to a wider distribution over different flocks. It is an important message for plant production that microbial interconnectivity will exist between ecosystems and that human pathogens can circulate between animals and plants when animal manure is applied to soil for fertilization [6]. Water used for irrigation is another human pathogen source in agricultural production systems, especially when derived from surface water bodies [7]. Human pathogens can contaminate surface water via drainage from arable fields recently fertilized with animal manure [8], but also from sewage overflow after severe precipitation [9] and wildlife [10]. 

The contamination of plant-derived products with human pathogens thus does not only result from harvest and post-harvest handlings, but can also occur at the primary production stage. The network activities of the EU COST Action on the control of human pathogens in plant production systems (HUPLANTcontrol) comprehended important aspects that were intended to gain a better understanding on the role of human pathogens in plant microbiomes in relation to ecology, taxonomical identity, and presumed virulence to humans. This information was relevant for the formulation of recommendations and guidelines to growers, but also to provide public information on the consequences of the presence of human pathogens in plant production systems. This Special Issue was dedicated to the main objectives of our network activities and resulted in seven manuscripts that are related to the topic of human pathogens in their relationship with plants.

It was shown that *Escherichia coli*, introduced via manure and seeds in production systems, had a higher preference for the root zone (roots and rhizosphere soil) than for the above-soil compartments [11,12]. Although different *E. coli* strains were incidentally found in stem parts shortly after their introduction, their abundance rapidly declined to levels below detection, whereas near, on, and inside roots, the introduced strains remained present up to plant senescence. As both experiments were performed under field-realistic circumstances, the key message derived from both manuscripts is critical for practice, because it would imply that plant roots are potential carriers of human pathogens once they are disseminated into production systems via external sources. The ability for microbial species to jump over from plant to animal kingdoms was indicated for two taxonomically distinct micro-organisms, *Fusarium musae* [13] and *Bacillus cereus* [14]. Namely, *F. musae* strains with the same genetic profile could infect both humans and plants (banana fruit), whereas *B. cereus* strains derived from 17 different agricultural soils sampled across Europe possessed genes that are potentially involved in human pathogenicity. Both studies made clear that human pathogens in plant production systems do not necessarily originate from external sources, but can be intrinsic members of soil and plant ecosystems. Soil treatment with composted sewage sludge resulted in a shift in the soil microbiome composition [15]. *Salmonella enterica* survived longer when simultaneously applied with composted sewage sludge to soil than when applied separately via irrigation. Changes in microbiomes as a result of soil amendments may thus influence the persistence of human pathogens in food production soils, and this information is relevant for understanding the mechanisms behind the soil persistence of human pathogens. Finally, it revealed that plants themselves can influence the behavior of human pathogens. Upon plant inoculation, flagellin expression was down-regulated in a vast majority of *S. enterica* cells, whereas high expression was found in a subfraction of the introduced population [16]. Heterogenous flagellin expression is an adaptational strategy of *S. enterica* inside plants. Plants defend themselves upon colonization by human pathogens via activating defensive networks [17]. Bioactive compounds produced by plants antagonize human pathogens in plants, offering new opportunities for the control of human pathogens in plant production systems.

The seven manuscripts in this Special Issue provide new and important information on the ecological behavior of human pathogens in the plant–soil environment and the roles that microbiomes play. They also demonstrated that plant microbiomes themselves harbor species that can potentially cross plant–animal frontiers and that the plant environment is a specific ecosystem where human pathogens are able to adapt to local prevailing circumstances. Valuable information was provided for further translation into practical recommendations, which is needed for the control of human pathogens in, or nearby, growing plants. Finally, the information provided is relevant for the transition towards extensive and circular agricultural production systems. The use of animal manure and other organic waste streams and reclaimed water as alternatives for fertilizers and irrigation water will become more opportune in this transition, affecting the introduction of human pathogens into plant production systems.
